# Design and Evaluation of Interprofessional Training Program for Healthcare Students from Collectivistic Culture

**DOI:** 10.1007/s40670-022-01536-7

**Published:** 2022-03-26

**Authors:** Sonika Raj, Dervla Kelly, MuizzI Siddig, Pranjali Muppidi, Chris O’Connor, Helena Mckeague, Mark Dixon, Mastour S. Alshahrani, Amani Alhazmi, Khalifa Elmusharaf

**Affiliations:** 1 Public Health Master Programme, University of Limerick, Limerick, Ireland; 2 School of Medicine, University of Limerick, Limerick, Ireland; 3 King Khalid University, Abha, Saudi Arabia; 4 Health Research Institute, University of Limerick, Limerick, Ireland

**Keywords:** Interprofessional education, Collectivistic culture, Healthcare students, Multidisciplinary learning

## Abstract

**Background:**

Healthcare is team-based, and with increased mobility of healthcare workers, most of them will work with team members from all over the globe. Interprofessional education (IPE) research has mostly focused on specially designed programs in academic health institutions to prepare students for multidisciplinary work. Few IPE programs aim to integrate students with mixed disciplines from collectivist cultures.

**Methods:**

This mixed-methods study was conducted between June and August 2019. Surveys and an e-portfolio were recorded of 33 final-year and graduated health professional students’ participation in an 8-week IPE summer program at a medical school in Ireland. Survey results are described, and the content of portfolios was analyzed based on the deductive analysis of qualitative data derived from questions.

**Results:**

Students reported the greatest improvement in presentation skills (63.6%), followed by communication (54.5%), team working skills (93.9%), and interprofessional learning (42.4%), respectively. Qualitative findings highlighted challenges for students from a collectivist culture adapting to an IPE: uncomfortable verbally expressing themselves in problem-based learning (PBL) and how to work with other sex. Positive themes about IPE that emerged were enjoyment in sharing ideas and building trust with PBL groups. We learned that the program had to be flexible enough to meet the educational requirements of a target community with mixed English language ability and adaptability to IPE.

**Conclusion:**

The authors propose that an international PBL-based summer program is effective in improving healthcare students’ attitudes towards IPE. This study provides valuable insights to facilitate the development of further IPE programs to increased collaboration between students across various healthcare disciplines.

**Supplementary Information:**

The online version contains supplementary material available at 10.1007/s40670-022-01536-7.

## Introduction

Over the past 50 years, interprofessional education (IPE) has been playing an increasingly significant role in healthcare training. As defined by the World Health Organization, IPE requires students from two or more professions to learn about, from, and with each other to enable effective collaboration and improve health outcomes [1]. This form of education works towards developing a common framework while preserving the competencies specific to each profession. It creates an environment for students to compare and contrast each other’s roles, responsibilities, and relationships and learn how one’s profession complements the others. It focuses on developing competencies in communication, collaboration, conflict management, and shared decision-making, all of which are skills needed to work within an interdisciplinary team [2, 3]. The skills students learn through this process are ones that they can apply and develop throughout their life, providing an opportunity for lifelong learning. Studies have also found that IPE-trained professionals were more cost-effective during their practice as they promoted the use of the most effective resources while preventing the use of redundant service [4]. Overall, IPE provided healthcare professionals an increased job satisfaction, reduced professional tensions, and helped facilitate better conflict management [4].

However, many factors contribute to the implementation of interprofessional education across countries. For instance, a person’s cultural identity as a collectivist or individualist influences the extent to which they are an independent learner [5]. This is because students in a collectivist society like those from the Middle East believes that knowledge is always transferred from an expert (teacher) to a learner (student), whereas students in an individualistic society are responsible for their learning while their teachers act as guides [6]. Educational practices in schools and universities in the Middle East do more to perpetuate dependency than to create self-direction [7]. This practice is further emphasized by a report describing the “spoon-feeding learning model” practiced by students and teachers in Oman [8]. Studies have shown that when students from a collectivist country are taught in cultural frameworks opposite from theirs, there was a higher incidence of depression, anxiety, and dependent personality traits [9]. Another factor to consider when changing learning styles is how a student from an autocratic country perceives the power relationship between a student and teacher differently from one who comes from a democratic country [5]. All these factors emphasize the importance of introducing scaffolds and developing strategies that facilitate students coming from a wide variety of backgrounds [10].

Interprofessional education can be delivered to students in different ways, one of which is problem-based learning (PBL). Problem-based learning uses problems and patient-based cases to direct students towards the learning outcomes. While solving the problems or cases, students have the opportunity to collaborate in an application-based scenario [11]. Self-directed learning (SDL) is a key aspect of PBL as this method provides learners the opportunity to work independently and take responsibility for the knowledge they acquire [12]. Problem-based learning help students become motivated learners and effective collaborators while developing a strong knowledge base, self-directed, and problem-based learning skills [13]. These aspects of PBL make it very suitable for IPE, and it has been shown that PBL use in IPE helps establish trust and respect between different disciplines [14, 15].

Given the globalization of the healthcare workforce, intercultural competency has become an important skill for team-based medical care. Cultural competence in models of IPE has largely focused on differing cultures associated with distinct disciplines [16]. Developing cultural awareness concerning racially, ethnically, culturally, and linguistically diverse groups, along with having an appropriate cultural sensitivity, is very important. Cultural sensitivity is defined as the concept of being “sensitive to the ways in which community members’ values and perceptions about healthcare differ from his or her own” [17]. Integrating these competencies in healthcare interprofessional education is important because it helps students understand how social and cultural factors influence a patient’s health beliefs and behaviors. Student participation in international interprofessional clinical placements has been shown to enhance cultural awareness, decrease ethnocentrism, and improve communication skills [18].

Engaging unprepared international students into modern Western health professional curricula remains one of the challenges for those who are involved in higher education teaching. Uncertainty, tradition, and hierarchy associated with collectivist culture have been shown to impact self-directed learning, thus impeding the uptake of the new learning approach [19]. It is also a challenge for international institutions to deliver a modern curriculum with a focus on learner autonomy to attract international students. This paper aims to describe the design and evaluation of an interprofessional education program delivered in Ireland to students from Saudi Arabia to promote their level of self-directed learning and lifelong learning skills for their future careers.

## Materials and Methods

### Participants and Study Design

This mixed-methods evaluation study included 33 Saudi Arabian health professional students who participated in an IPE training program conducted for 8 weeks (June and July 2019), at the School of Medicine, University of Limerick, Ireland. The students were from public health (8), medical rehabilitation (7), radiology (7), clinical laboratory (7), and dental technology (4) disciplines. These students were selected by the Saudi Arabian University based on their academic performance and willingness to participate in the study. Twenty students were males and 13 were females.

### Design of Curriculum

This IPE training program aimed to foster learning and collaboration among healthcare students to enhance self-directed learning and promote their lifelong learning. To achieve this, the program employed a variety of learning methodologies such as problem-based learning, self-directed learning, teachers as role models, educational field trips, and reflective e-portfolio.

The contact theory by Allport (1954) was used to design the IPE training program [20]. According to him, positive interprofessional collaboration requires not only bringing the different groups together but also meeting the following four conditions:

1. *Equal status between different groups*: to meet this criterion, all the students were allocated in four multi-professional and mixed sex groups of 8 to 9 students each, following an introductory session focusing on PBL, SDL, and lifelong learning. Each group was facilitated by an expert PBL facilitator.
2. *Groups should work on common goals*: the four cases for problem-based learning were based on the main causes of mortality in Saudi Arabia [21]. The four cases were cardiovascular diseases, diabetes, road injuries, and major emergencies. Each problem-based case lasted for 1 week during which each group of students met for 8 h to discuss the case of that week. The weekly timetable had protected time for self-directed learning. Students were instructed to use the protected time to focus on their learning objectives.
3. *Groups should cooperate and not compete with each other*: the interprofessional tasks were designed in such a way that required the combined knowledge and cooperation of professional groups and facilitate a mutual understanding among them. The four PBL cases lasted for 4 weeks. For another 2 weeks, students from each discipline were asked to work together to design a discipline-specific case that could be used for a future PBL session. Students then acted as facilitators for PBL sessions to students from other disciplines.
4. *The program should have the support of authorities*: a team of experts from Public Health, Medical Education, Paramedics, and Allied Health at the University of Limerick in Ireland and King Khalid University in Saudi Arabia worked together to design this IPE training program. National and international experts from various backgrounds delivered series of lectures, current perspectives, and experiences on interprofessional and collaborative practice, healthcare research methods, good clinical practices, empathy in healthcare, professionalism, and cultural competency.

During the program, students were engaged in numerous outdoor high-fidelity simulation exercises of a multi-casualty incident requiring a multidisciplinary, inter-agency response. They also engaged in performing a variety of team-building exercises. They conducted various field visits to University Hospital Limerick, Clinical Education and Research Centre, Limerick Fire and Rescue Services, and Coast Guard Helicopter Rescue Service. These field visits aimed to allow students to observe and compare the organization, structure, and function of health systems across international settings.

Since the students were from a non-English speaking country, an English module was designed and delivered to them to build their capacity across the key language skills—written, spoken, comprehension, and interaction. Students were trained on report writing, writing a proposal, and a discursive essay. The reading and listening components included a variety of texts incorporating articles and interviews along with promotional, informational, and authentic materials. The speaking component focused on collaborative communication tasks with students actively engaging in pair and group discussions.

Students also used an e-portfolio weekly to reflect on their experiences.

### Evaluation

This study involved multi-method evaluations of the training program that included qualitative and quantitative analysis of participants’ satisfaction, self-reported changes in knowledge, skills, competencies, and experiences:

1. *Pre and post-English language test*: students undertook the English Oxford placement test at the beginning of the program. This gave them a score out of 100 and also gave a CEFR mark (Common European Framework of Reference). This is an international standard for describing language ability. It describes language ability on a six-point scale, from A1 for beginners, to C2 for those who have mastered a language. The facilitator then placed the students in the relevant groups according to their scores [22]. At the end of the course, the students were again assessed and were given feedback on their results.
2. *Self-assessment of knowledge, skills, and competencies (Appendix *1*):* for self-assessment of skills like presentation, communication, writing, team working, crisis management, language skills, and interprofessional learning after the training program, a scale of 0–4 from “no improvement to high improvement” was used. For assessment of improvement in self-directed learning, lifelong learning, and intercultural learning, three respective standardized questionnaires were used as such without modifications, 20 questions on SDL [23], 15 questions on Intercultural competence [24], and 30 questions on lifelong learning [25], respectively. The mean score was calculated before and after the training program.
3. *E-portfolio (Appendix *2*):* the e-portfolio of the students was also analyzed to have insight into their experiences in a new country, working in a multidisciplinary group with mixed sex, problem-based learning approach, and intercultural competence.

### Data Analysis

Quantitative data were analyzed using IBM SPSS Statistics for Windows, version 26.0, and presented as means, standard deviation, and frequencies. Paired sample *t* test was used to compare the mean differences among the scores before and after the training.

Qualitative data were analyzed using thematic content analysis. A deductive approach was taken with predefined themes identified based on e-portfolio questions. Three researchers independently familiarized themselves with the data, categorized it by identifying meaningful and relevant quotes; placing the quotes under the appropriate category; and meeting to agree on their interpretation of the quotes iteratively to reach an agreement on the key themes that emerged.

### Ethical Considerations

Informed consent was taken from the students for participation in the study. Data were anonymized with each participant assigned a code to use for survey responses which were used on the data entry sheet. Ethics approval was obtained from the Institutional Review Board, University of Limerick (Ethics approval number 20190628EHS).

## Results

### Participants Characteristics

A total of 33 students from five healthcare professions attended the program with the majority of males (60.6%) and in their final year (66.7%) of professional degree (Table 1).

**Table 1:** Characteristics of students

**Variables**	**Frequency (*n*** **= 33)**	**Percentage**
**Gender**		
Male	20	60.6
Female	13	39.4
**Year of college**		
Final year	22	66.7
Graduated	11	33.3
**Discipline**		
Dental technology	4	12.1
Medical laboratory sciences	7	21.2
Medical rehabilitation	7	21.2
Public health	8	24.2
Radiology	7	21.2

### Self-Assessed Improvement in Skills

Students reported the greatest improvement in presentation skills (63.6%), followed by communication (54.5%) and team working skills (54.5%), and interprofessional learning (42.4%) respectively. Moderate improvement was found in writing (72.7%) and crisis management skills (69.7%) as shown in Table 2. The majority of students (84.8%) noticed moderate to high improvement in English language skills.

**Table 2:** Self-assessed improvement of students in various skills after the program

*N* = 33	Writing skills *n* (%)	Communication skills *n* (%)	Presentation skills *n* (%)	Team working skills *n* (%)	Interprofessional learning *n* (%)	Crisis management *n* (%)	English language skills *n* (%)
High improvement	5 (15.2%)	18 (54.5%)	21 (63.6%)	18 (54.5%)	14 (42.4%)	3 (9.1%)	10 (30.3%)
Moderate improvement	24 (72.7%)	13 (39.4%)	11 (33.3%)	13 (39.4%)	18 (54.5%)	23 (69.7%)	18 (54.5%)
Slight/no improvement	4 (12.1%)	2 (6.1%)	1 (3.0%)	2 (6.1%)	1 (3%)	7 (21.2%)	5 (15.2%)

The mean score of the participants increased in all the competencies after the program, and the difference was found to be statistically significant (*p* < 0.001). The mean English language score increased statistically significantly from 39.5 ± 26.7 to 71.1 ± 13.2 after the training (*p* < 0.001) (Table 3).

**Table 3:** Mean score of students in various competencies after the program

	Before Mean (SD)	After Mean (SD)
Self-directed learning score	3.4 (0.6)	4.3 (0.5)^**^
Lifelong learning	3.4 (0.5)	3.9 (0.6) ^**^
Intercultural competencies	3.3 (0.5)	4.2 (0.4) ^**^
Group work	3.4 (0.7)	3.6 (0.8) ^**^
English language score	39.5 ± 26.7	71.1 ± 13.2^**^

^**^ *P* < 0.01

Overall, students’ perceptions of the program were also consistent with our analysis and stating that the problem-based learning was beneficial and they would recommend the program to other students in their University as indicated by the following quotes:

 “*I had a great experience from this training. I believe that self-directed learning is THE BEST way to gain information or to build better knowledge”.* Student 5.

 “*I learned a lot of things but I feel that the most important for me is the problem-based learning, and I think it will helpful to be a lifelong learner. I will recommend this training program to all my colleagues”.* Student 18.

 “*It was amazing communicating with people with a different culture, accepting the other, improving the skills of presentation, improving the English language and learning some new things from different disciplines”*. Student 16.

#### Qualitative Analysis of Experiences of Students About the Program Captured Through an E-Portfolio

We identified five themes in the qualitative data: (1) adapting to PBL, (2) recognizing cultural differences, (3) working in multidisciplinary teams, (4) working with different sex, and (5) the experience of living away from home:

1. *Adapting to PBL as a learning method*: at the beginning of the training program, some of the students found it difficult to cope with the PBL approach due to their unfamiliarity with this method. Participants discussed the prolonged period PBL sessions take to deliver information in contrast to the traditional method of learning. Another challenge they encountered during the first few sessions of PBL was the lack of communication between group members as they were inactive during discussions. Some of the students attributed it to their feelings of shyness and nervousness accompanied by a lack of confidence.
  However, at the end of the training program, most of the students were able to adapt to the PBL technique. They described it as an exciting and productive approach to acquire knowledge and become a self-directed learner. Being independent to search for information and share it with their classmates were intriguing factors for them to apply this technique in their future careers. Moreover, they conveyed how PBL can foster their confidence and encourage them to be an effective member when working in teams. Most of them recommended applying this method in their home county as a part of their curriculum to achieve better learning outcomes:
  *“This week I felt mad all time, I need more things to learn and I don’t like to search in google because I do not know from where to get the correct information.”* Student 10, week 3.
  *“At first, I was anxious and very nervous, and we did not get the idea (of PBL) fast so I was refusing the PBL but after some classes, I became more confident and started to enjoy the PBL classes and excited to learn new information.”* Student 27, week 4.
  *“In the beginning, the PBL was confusing and I didn’t like it… I thought the traditional teaching is better but now I think the PBL is a good way to learn. The traditional teaching is boring, but it is a quicker way to get information while the PBL approach does not give direct answers, so it takes a long time to get the subject.”* Student 17, week 4.
  *“My PBL experience included many good skills as I learned how to work with the group, organize interpersonal dialogue, create the right environment for discussion, try to correct mistakes among members and cooperate among them.”* Student 5, week 5.
  *“I learned to rely on myself, think correctly, also learned that you must plan before everything. It helped me to define where my strengths and weaknesses are. I will apply everything I learn and know to solve problems and overcome crises in the future. I also developed my English.”* Student 28, week 6.
2. *Recognizing cultural differences*: students appreciated the opportunity to think about their own cultural identity and norms and entire IPE experience. It encouraged them to think about differences within cultures and learning expectations and the importance of respecting differences between and among cultural groupings. The students faced some challenges including unsatisfactory food, transportation to and from the university, and cold Irish summer weather compared to their home country:
  *“I developed cultural self-awareness, learned to appreciate and value diverse views, avoiding imposing own values on others, resist stereotyping.”* Student 3.
  *“Learnt how to respect the culture of others and how to live with it, everyone has a point of view that responds respect.”* Student 31.
  *“I learnt about Cultural Interaction and how can we communicate effectively with people from different cultures and about cross-cultural interactions.”* Student 19.
3. *Working in a multidisciplinary team*: the students described working in a multidisciplinary team as a new experience where they developed their capabilities and boosted their confidence. They enjoyed sharing ideas and thinking collectively about the delivery of healthcare services. They reported gaining new information and insights from their peers and valued each other’s opinions and perspectives. A minority of students preferred to work solely with peers from their specialty. They felt that working within the scope of their discipline allowed them to advance to a deeper level of specialty knowledge:
  *“Sharing my ideas helped me to become more confident and a better speaker and I think it’s same for others.”* Student 27.
  *“Working and learning with a group of sex and also from different disciplines opens your mind, expands your perceptions, and gives you a comprehensive picture of the subject.”* Student 6.
  *“This increased the communication between me and my coworkers and enhanced our productivity as a team. It helped us to delegate tasks more easily. Everyone brought their skills, talents, and experiences together for a common goal. I enjoyed brainstorming and collaborating with my colleagues.”* Student 11.
  *“Initially there was lack of cooperation among group members and had different views but with time everyone started respecting each others’ differences and opinions.”* Student 15.
  *“The program is not useful in developing our skills in our specialties. We want Medical Clinics and cases to practice our specialties.”* Student 13.
  *“PBL groups are mixed with other members from different specialties. What I wanted was the PBL groups should be at least divided according to students’ specialties.”* Student 3.
4. *Working with different sex*: working with different sex in an educational setting was a new and uncomfortable experience for most of the participants. However, gradually over time, most participants reported feeling more comfortable with each other, and there was improved cooperation during classes. Moreover, they discussed how working with the other sex could strengthen their confidence and prepare them to work effectively in their future career. Contrary to the belief, facilitators observed that female students were taking lead in all group work and case presentations:
  *“At the beginning, it wasn’t easy to collaborate and work together to achieve a certain goal, but after 4 weeks of working with the other sex, I find it much easier and simpler. Even the other gender started to become looser when it comes to working with us. I think it’s a matter of time and respect.”* Student 33, male.
  *“Working with other sex was a new experience for us, but over time I realized that it is better because you will get to know their opinions and will prepare to work in the outside world in future.”* Student 2, female.
  Notably, some females conveyed how working with males is undesirable and they prefer to collaborate only with their sex. They attributed their feelings to their cultural values and preconceived ideologies that males hold towards them:
  *“It was different to be in class together. Actually, not all men are the same, some of them (are) frustrating to work with. They are not helpful at all and they look down to us as women.” *Student 28, female.
  *“Study with a different sex makes me feel strange and uncomfortable because I do not like (to) work and study together because we women think about a completely different way, we do not have any common interest.”* Student 9, female.
5. *Experience of living away from home*: all participants were away from their friends, family, and country for the first time. They had mixed feelings. At one end, they were happy to explore the new places, traditions, customs, and lifestyles of a new country. They learned to become independent, made friends, and adapted to the new environments. But, at the same time, they were missing their families and friends back home. Some of them wish to come back again for such a training program:
  *“It was a great experience to be out of my country for a while. I discovered new traditions and a new lifestyle but I’m also missing my family and my friends. I want to go back but I also want to stay here. I’ve new friends now and they are amazing. I will transfer the experience to my friends.”* Student 32.
  *“It was an unforgettable and important experience for my personal and professional life. I learned to become independent. I would be happy to come back again.”* Student 7.

## Discussion

The study focused on the evaluation of the IPE training program delivered to students from the collectivistic culture in Ireland using a multidimensional design. A hybrid PBL method was used for the delivery of training with other program components including lectures, tutorials, interactive sessions, case studies, field visits, use of e-portfolio, peer learning, and skills training. It has been hypothesized that the collectivist culture of the students may make PBL-type interactions or discussions challenging [23]. Some students were resistant to the exploration and discovery style of PBL teaching saying it was too time-consuming. However, overall, our study found most of the students demonstrated an increase in teamwork and language skills during the program, suggesting that despite never previously working in PBL tutorials, the PBL teaching strategy was effective in achieving the objectives of the program.

Students indicated that PBL encouraged them to self-assess their knowledge and understand the challenges and rewards of working in multidisciplinary teams, improved their communication skills, and prepared them to undertake more self-directed learning after they completed the course. The findings are in agreement with the benefits of PBL style teaching in IPE literature which has been highlighted in other settings [14, 26, 27].

It has previously been reported that culture can strongly influence the development of self-directed learning readiness [28]. We found conflicts frequently arose in the minds of students from Saudi Arabia when comparing their cultural norms of unidirectional knowledge transfer from teachers to students to the expectations of our PBL-based IPE program which requires an active participation of students with teachers only acting as facilitators [6, 7]. Being shy and uncomfortable questioning information in PBL was reported in the qualitative results. In addition, the new experience of working with a different sex for the first time was challenging and uncomfortable, especially for females. The themes of student anxiety overexpressing uncertain ideas and females avoiding collaboration with males due to their cultural differing roles have also been reported elsewhere [29, 30]. We found they gradually adjusted, and surprisingly, we found that female students were taking lead in all group work and case presentations. In Saudi Arabia, although increased participation of women in the workforce is being encouraged, sex segregation is a cultural practice that occurs across all public and private domains.

In terms of evaluating the effectiveness of the IPE aspect of the program, the literature reports three main levels of changes: changes in attitudes and perceptions of students, acquisition of knowledge of other healthcare professionals’ roles and development of collaborative skills, and change in perceived or actual collaborative behavior [31]. Our study found evidence of the first two levels of changes, most notably in the qualitative e-portfolio where sharing and building trust and respect were described by the students. Interestingly, with this Saudi cohort, the acquisition of knowledge of other disciplines and discovering an appreciation for multidisciplinary learning were similar regardless of the student’s discipline. Few studies compare changes in attitudes across professions, and the literature suggests that the perceived need for cooperation and consequently IPE varies between professionals [32, 33].

It is also useful to reflect on what we learned in terms of developing and implementing the program. The majority of the students were dissatisfied with the non-profession-specific tutoring and wanted more discipline-specific guidance. We learned that setting expectations around the learning objectives of IPE is important to prevent students from getting frustrated when developing specialist knowledge is not the objective of a lesson. Given the hesitance of some students to working with other professions and different sex, we recommend sensitizing the students regarding IPE before they arrive on placement. These issues need to be taken into account for organizations looking to run similar programs. Further research is also required to determine whether any of the changes in knowledge and attitudes found in this study impact future practice and, which, if any, can be attributed to the IPE training program. Organizational issues in conducting this 8-week residential training program cannot be underestimated.

The strength of the study is the novel design with students from a collectivist culture receiving IPE training in a western country. The study has some limitations. The evaluation of the program was mainly based on the self-assessment of the students. However, literature has shown that the development of interprofessional knowledge and skills among students has mainly been evaluated through self-reported changes in understanding and performance [26, 34]. Secondly, the students in this study had no/minimal exposure to IPE training, so their application of knowledge may only come with further such learning experiences, and it is difficult to assess these outcomes immediately after the program. We are planning for a follow-up of the study to explore student’s perspectives about the outcomes of the training program and to evaluate the impact of the training program on their self-directed and lifelong learning knowledge and skills. Thirdly, since the training program was conducted in the summer and due to limited resources, the students from Saudi Arabian University could not mix up with local Irish students or students from other countries; however, the students were exposed to Irish culture, games, food, dance, and music.

## Conclusion

We used a multidimensional approach used for designing and evaluating a pilot study of an IPE training program. We found an international PBL-based IPE program was effective in developing self-directed and lifelong learning skills and teamwork skills required to work in a multi-professional team of healthcare professionals. The success of the program suggests that PBL was an appropriate choice of educational strategy, which emphasized active participation of students through interprofessional small-group work and context-specific, clinically based cases and feedback, where appropriate. Qualitative findings highlighted challenges for students from a collectivist culture adapting to a PBL-based IPE: uncomfortable verbally expressing themselves in PBL and how to work with other sex. Positive themes about IPE that emerged were enjoyment sharing ideas and building trust with PBL groups. This study provides valuable insights to facilitate the development of further IPE programs to increased collaboration between students across various healthcare disciplines. Considering the usefulness and need of interprofessional training for healthcare professionals in the current scenario, there is a need to scale up such training programs.

## Supplementary Information

Below is the link to the electronic supplementary material.

Supplementary file1 (DOCX 23 KB)

## Data Availability

All data generated or analyzed during this study are included in this published article.

## Abbreviations

IPE: Interprofessional education
PBL: Problem-based learning
SDL: Self-directed learning
UL: University of Limerick
CEFR: Common European Framework of Reference

## Declarations

### Author Information

Sonika Raj is a postdoctoral researcher at the Public Health Master program at the University of Limerick. Dervla Kelly is a lecturer in medical education at the University of Limerick, Medical School. Muizz Siddig is a doctoral student and research assistant at the Public Health Master program at the University of Limerick. Pranjali Muppidi is a medical student at the University of Limerick, Medical School. Chris O’Connor is a Clinical Tutor at Paramedic Studies, the University of Limerick. Helena Mckeague is a senior lecturer PBL and BMBS Course Director at the University of Limerick, Medical School. Mark Dixon is a course director, paramedic studies, Medical School, University of Limerick. Mastour S. Alshahrani is a Chair of the Department of Medical Rehabilitation Sciences, King Khalid University, Saudi Arabia. Amani Alhazmi is an Assistant Professor at the Department of Public Health and a Vice Dean in Applied Medical Sciences College, Saudi Arabia. Khalifa Elmusharaf is a senior lecturer in public health and the director of the Public Health Master program at the University of Limerick.
