# Supplementary material for: Design and Evaluation of Interprofessional Training Program for Healthcare Students from Collectivistic Culture
Source: Med Sci Educ. 2022 Mar 26;32(2):447–55. doi: 10.1007/s40670-022-01536-7 (PMC9054974; doi:10.1007/s40670-022-01536-7)
Supplement: Supplementary file 1 — Supplementary file1 (DOCX 23 KB) [file 40670_2022_1536_MOESM1_ESM.docx]

**Appendix A**

**Questionnaire for Survey**

|  | **Section and question** | **Answer Type** |
| --- | --- | --- |
| **Demographics** | |  |
|  | Name |  |
|  | KKU student ID |  |
|  | Course |  |
|  | Year of Graduation |  |
|  | | |
| **Group/Project Work questionnaire** | |  |
| A | I like to do several activities at the same time. | Strongly Agree, Somewhat Agree, Neither Agree nor Disagree, Somewhat Disagree, Strongly Disagree, Don't know, Refused |
| B | I would rather complete an entire project every day than complete parts of several projects. | As (a) |
| C | I believe people should try to do many things at once. | As (a) |
| D | When I work by myself, I usually work on  one project at a time. | As (a) |
| E | I prefer to do one thing at a time. | As (a) |
| **Lifelong learning preferences** | |  |
| **After the 8 weeks program, when I want or need to learn something I will:** | |  |
| a | find someone to teach me who knows about the topic, such as a friend, co-worker, family member | Always, Most of the time, Some of the time, Never, Don’t know, Refused |
| b | take classes or attend community seminars or workshops about what I want to learn | As (a) |
| c | look for educational or informational television or radio programs about what I want to learn | As (a) |
| d | read newspapers articles, magazine articles, books, or journals on the topic I want to learn about | As (a) |
| e | Search the Internet for information about what I want to learn | As (a) |
| f | buy, rent, or borrow audio or videotapes about what I want to learn | As (a) |
| g | start by doing what I want to learn about and learn from my mistakes and successes | As (a) |
| h | find a public event, exposition, or exhibition featuring what I want to learn about | As (a) |
| i | enroll in a college or community college to take classes about what I want to learn | As (a) |
| j | find a tutor or professional to teach me what I want to learn about | As (a) |
| k | find an online or chat group interested in a topic I want to learn about | As (a) |
| l | get involved in a community group or volunteer organization that focuses on what I want to learn about | As (a) |
| **Life long learning preferences:**  **These next statements describe why you want to learn.** | | Strongly Agree, Somewhat Agree Neither Agree nor Disagree Somewhat Disagree, Strongly Disagree, Don't know, Refused |
| a | so I can improve my job skills to make work easier or to get ahead | Strongly Agree, Somewhat Agree, Neither Agree nor Disagree, Somewhat Disagree, Strongly Disagree, Don't know, Refused |
| b | so I can earn a degree or certification that will advance my career or help me earn more money | As (a) |
| c | so I can I enjoy my hobbies or recreational activities better | As (a) |
| d | so I can manage my everyday life better | As (a) |
| e | so I know the basic skills I need every day, such as reading, writing, basic math, or learning how to drive | As (a) |
| f | for the simple joy of learning something new | As (a) |
| g | so I can give back something to my community | As (a) |
| h | so I can understand myself better | As (a) |
| i | so I can understand and get along with others better | As (a) |
| j | so I can help other people | As (a) |
| k | so I can keep up with what’s going on in the world | As (a) |
| l | so I can talk about things with my children or parents OR friends | As (a) |
| m | for my spiritual or personal growth | As (a) |
| I learn best... | |  |
| a | in a group where I can share with other learners | Strongly Agree, Somewhat Agree, Neither Agree nor Disagree, Somewhat Disagree, Strongly Disagree, Don't know, Refused |
| b | when I have a guide, instructor, tutor, or mentor in a face-to-face situation | As (a) |
| c | by putting my hands on something and playing with it or figuring it out | As (a) |
| d | by watching or listening and then thinking about it | As (a) |
| e | in a formal teacher-classroom situation | As (a) |
| f | by gathering as much information as I can find and then teaching myself | As (a) |
| g | by finding self-study courses that let me set my own objectives and then moving at my own pace. | As (a) |

How would you rate your improvement in lifelong learning skills after the 8 weeks programme (scale 0-10)

**Self-Directed Learning Questions**

*Directions*: Please read each statement and indicatethe number that best describes your thoughts and feelings about your own learning after **the 8 weeks program**. There is no right or wrong answer.

5 = Strongly agree, 4 = Agree, 3 = Neutral, 2 = Disagree, 1 = Strongly disagree

| 1 | I know what I need to learn. | 5 | 4 | 3 | 2 | 1 |
| --- | --- | --- | --- | --- | --- | --- |
| 2 | Regardless of the results or effectiveness of my learning, I still like learning. | 5 | 4 | 3 | 2 | 1 |
| 3 | I strongly hope to constantly improve and excel in my learning. | 5 | 4 | 3 | 2 | 1 |
| 4 | My successes and failures inspire me to continue learning. | 5 | 4 | 3 | 2 | 1 |
| 5 | I enjoy finding answers to questions. | 5 | 4 | 3 | 2 | 1 |
| 6 | I will not give up learning because I face some difficulties. | 5 | 4 | 3 | 2 | 1 |
| 7 | I can pro-actively establish my learning goals. | 5 | 4 | 3 | 2 | 1 |
| 8 | I know what learning strategies are appropriate for me in reaching my learning goals. | 5 | 4 | 3 | 2 | 1 |
| 9 | I set the priorities of my learning. | 5 | 4 | 3 | 2 | 1 |
| 10 | Whether in the clinical practicum, classroom, or on my own, I can follow my plan of learning. | 5 | 4 | 3 | 2 | 1 |
| 11 | I am good at arranging and controlling my learning time. | 5 | 4 | 3 | 2 | 1 |
| 12 | I know how to find resources for my learning. | 5 | 4 | 3 | 2 | 1 |
| 13 | I can connect new knowledge with my own experiences. | 5 | 4 | 3 | 2 | 1 |
| 14 | I understand the strengths and weaknesses of my learning. | 5 | 4 | 3 | 2 | 1 |
| 15 | I can monitor my learning progress. | 5 | 4 | 3 | 2 | 1 |
| 16 | I can evaluate on my own my learning outcomes. | 5 | 4 | 3 | 2 | 1 |
| 17 | My interaction with others helps me plan for further learning. | 5 | 4 | 3 | 2 | 1 |
| 18 | I would like to learn the language and culture of those with whom I frequently interact. | 5 | 4 | 3 | 2 | 1 |
| 19 | I am able to express messages effectively in oral presentations. | 5 | 4 | 3 | 2 | 1 |
| 20 | I am able to communicate messages effectively in writing. | 5 | 4 | 3 | 2 | 1 |

How would you rate your improvement in self-directed learning skills after the 8 weeks program (scale 0-10)

**Intercultural competence Questions**

*Directions*: The items listed below are invaluable in developing intercultural competence and in interacting effectively and appropriately with people from other cultures. Please rate yourself **after the 8 weeks program,**on each of the following:

5 = Very high, 4 = High, 3 = Average, 2 = Below average, 1 = Poor

| 1 | Respect (valuing other cultures) | 5 | 4 | 3 | 2 | 1 |
| --- | --- | --- | --- | --- | --- | --- |
| 2 | Openness (to intercultural learning and to people from other cultures | 5 | 4 | 3 | 2 | 1 |
| 3 | Tolerance for ambiguity. | 5 | 4 | 3 | 2 | 1 |
| 4 | Flexibility (in using appropriate communication style and behaviours, in intercultural situations) | 5 | 4 | 3 | 2 | 1 |
| 5 | Curiosity and discovery | 5 | 4 | 3 | 2 | 1 |
| 6 | Withholding judgement | 5 | 4 | 3 | 2 | 1 |
| 7 | Cultural self-awareness/understanding | 5 | 4 | 3 | 2 | 1 |
| 8 | Understanding others’ worldviews. | 5 | 4 | 3 | 2 | 1 |
| 9 | Culture-specific knowledge | 5 | 4 | 3 | 2 | 1 |
| 10 | Sociolinguistic awareness (awareness of using other languages in social contexts | 5 | 4 | 3 | 2 | 1 |
| 11 | Skills to listen, observe and interpret | 5 | 4 | 3 | 2 | 1 |
| 12 | Skills to analyse, evaluate, and relate | 5 | 4 | 3 | 2 | 1 |
| 13 | Empathy (do unto others as you would have others do unto you) | 5 | 4 | 3 | 2 | 1 |
| 14 | Adaptability (to different communication styles/behaviours, to new cultural environments | 5 | 4 | 3 | 2 | 1 |
| 15 | Communication skills (appropriate and effective communication in intercultural settings) | 5 | 4 | 3 | 2 | 1 |

How would you rate your improvement in intercultural competence after the 8 weeks program (scale 0-10)

**Evaluating PROGRAMME CONTENT & DELIVERY**

| **General Questions on the Summer Programme** | |  |
| --- | --- | --- |
|  | Briefly, what did you feel worked well during the summer school? | Free text |
|  | Briefly, what didn’t work well? | Free text |

**Appendix B**

**E-portfolio Questions**:

**Week 1 & 2:** Describe your experience of your first week in an unfamiliar country?

**Week 3:** Please reflect upon your experience of working in a multi-disciplinary group with mixed genders.

**Week 4:** Please compare your experience of the ‘Problem Based Learning’ approach to Teaching & Learning with your experience of ‘Traditional’ teaching methods.Also, discuss the input of your colleagues into the group work. How beneficial or helpful has it been?

**Week 5:**Please reflect upon how it feels to have been away from home for over 6 weeks. Is there anything/anyone you miss? Family & Friends? Have you been homesick? Or perhaps would you like to stay for longer?

**Week 6:**Please reflect upon lecture on ‘Inter-Cutural competency’and relate it to your personal experiences as International Students.
